# *Neisseria meningitidis* Serogroup W135, China

**DOI:** 10.3201/eid1602.090901

**Published:** 2010-02

**Authors:** Zhujun Shao, Haijian Zhou, Yuan Gao, Hongyu Ren, Li Xu, Biao Kan, Jianguo Xu

**Affiliations:** Chinese Center for Disease Control and Prevention, Beijing, People’s Republic of China

**Keywords:** Neisseria meningitidis W135, ST11, bacteria, China, letter

**To the Editor:** *Neisseria meningitidis* is a gram-negative bacterium found only in humans and is a major cause of serious invasive diseases. Before 2006, in the People’s Republic of China, all meningococcal diseases were caused by serogroups A, B, and C. However, there are >13 serogroups of this organism. Three cases of infection with *N*. *meningitidis* serogroup W135 were reported in China during 2006–2008. We describe these 3 meningitis patients and the *N*. *meningitidis* serogroup W135 strains isolated from these patients by genotyping methods.

Patient 1, a 36-year-old man, was seen at a local hospital in Fujian Province in January 2006. He became ill while on a business trip and was given a diagnosis by culture of an *N*. *meningitidis* infection. Patient 2, a 25-year-old man, was seen in Guangdong Province in May 2007. He had not traveled outside this area in the 10 days before becoming ill. Patient 3, a 14-year-old girl, was seen in Guangxi Province in February 2008. She was a middle school student and had toured the suburbs of this province with her classmates 2 days before becoming ill. Close contacts of all 3 patients were investigated; no additional *N*. *meningitidis* infections were detected. However, *N*. *meningitidis* was isolated from a throat swab specimen obtained from the younger cousin of patient 3.

*N*. *meningitidis* infection was confirmed for all 3 patients on the basis of clinical symptoms and laboratory results. All patients reported neck stiffness. Physical examinations showed Kernig signs, Brudzinski signs, and high temperatures (>38°C). Cerebrospinal fluid (CSF) samples were turbid with increased protein levels and pressure; leukocyte counts were increased (>5,000 cells/μL). CSF culture on chocolate agar grew *N. meningitidis* after 24 h. Isolates were identified as serogroup W135 by using specific antiserum (Remel, Lenexa, KS, USA) at provincial Centers for Disease Control and Prevention (CDC) in China and confirmed at the China CDC.

Patients were treated with antimicrobial drugs and recovered fully. An isolate from the cousin of patient 3 was also identified as W135. Etest strips and broth microdilution were used for antimicrobial drug susceptibility testing for the 4 W135 isolates. All isolates were susceptible to 12 antimicrobial drugs tested, which included therapeutic and prophylaxis agents used frequently in China.

Pulsed-field gel electrophoresis (PFGE), multilocus sequence typing, and outer membrane protein (*por*A) gene variant region subtyping were used to characterize the 4 case-related W135 *N*. *meningitidis* isolates and other isolates from asymptomatic carriers. Strain R29057 (from France) was used as a reference strain. The 4 case-related isolates showed similar PFGE patterns. These patterns were distinct from those of other W135 isolates obtained from asymptomatic carriers. Three invasive disease isolates and 1 from the close contact of patient 3 had the same multilocus sequence type (ST) and PorA subtype; all were ST11: P1.5, 2. This subtype was not detected among other tested isolates of W135 obtained from asymptomatic carriers (Technical Appendix).

ST11: P1.5, 2 *N*. *meningitidis* serogroup W135 was responsible for the epidemic of W135 meningococcal disease in 2000, which was associated with the Hajj pilgrimage in Saudi Arabia (*1**,**2*). The strain related to the Hajj pilgrimage was derived from clonal expansion within the ST11 complex/ET-37 complex (*3*). However, no epidemiologic data showed that the 3 cases in our study were linked to the Hajj pilgrimage. Since 2000, invasive diseases caused by W135 meningococci of ST11 have been reported in Africa, Asia, and the Middle East (*4*). ST11 W135 infections have been reported to cause invasive disease in Taiwan during 1996–2002 and were apparently introduced into Taiwan before the Hajj pilgrimage–associated outbreak because they were genotypically distinct from the Hajj-related W135 clone (*5**,**6*).

The 3 cases we report were observed in southeastern China near Taiwan (Technical Appendix), but no direct epidemiologic links are known. Because of the lack of W135 strains from Hajj pilgrimages and Taiwan in this study, we could not provide a detailed and integrated genotypic relationship between the strains in China and those of Hajj pilgrimages and Taiwan. However, we can confirm that these 3 cases were caused by strains from the same hypervirulent clone characterized as ST11: P1.5, 2.

W135 strains have been isolated after vaccination with a bivalent meningococcal vaccine in Cameroon (*7*). In China, the bivalent meningococcal vaccine has been successfully introduced into the national expanded immunization program in response to an outbreak of *N*. *meningitidis* serogroup C during 2003–2004 (*8*).The 3 patients infected with W135 in our study did not receive bivalent meningococcal vaccines. W135 meningococcal disease appears to be an emerging problem that should be investigated epidemiologically. These patients highlight the need for further epidemiologic surveillance to monitor changes in the incidence of meningococcal disease caused by W135 and for improved public health disease control strategies in the future.

## Supplementary Material

Technical Appendix Neisseria meningitidis Serogroup W135, China
