# Supplementary material for: Neisseria meningitidis Serogroup W135, China
Source: Emerg Infect Dis. 2010 Feb;16(2):348–9. doi: 10.3201/eid1602.090901 (PMC2958009; doi:10.3201/eid1602.090901)
Supplement: Technical Appendix — Neisseria meningitidis Serogroup W135, China [file 09-0901_Techapp-s1.pdf]

Neisseria meningitidis Serogroup W135, China

Technical Appendix

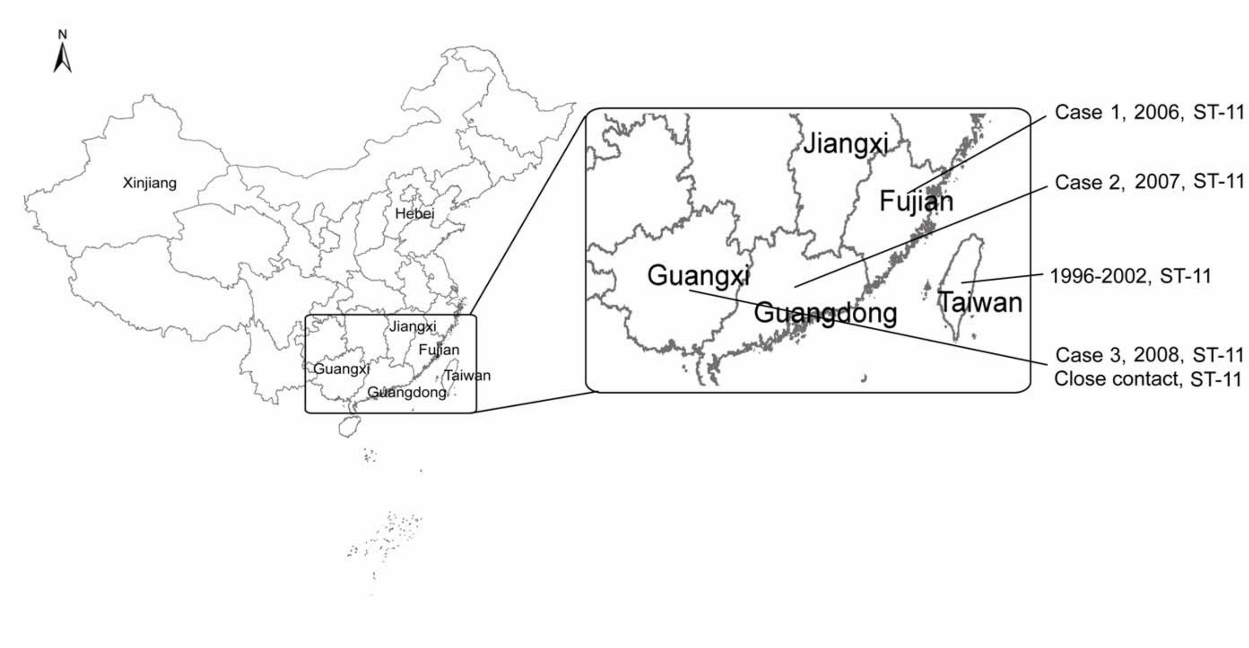

Figure 1. Distribution and molecular characterization of *Neisseria meningitidis* W135 isolates from 3 patients, People's Republic of China, and from Taiwan. ST, sequence type.

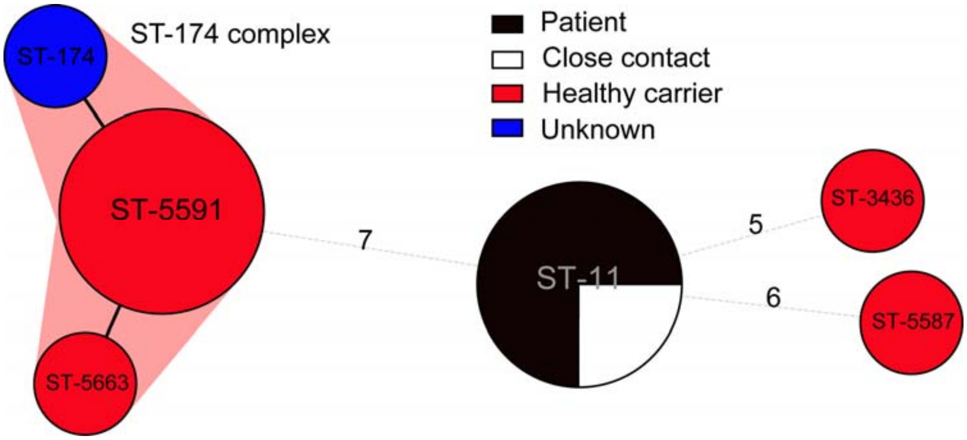

Figure 2. Distribution and molecular characterization of *Neisseria meningitidis* W135 isolates from case-patients, People's Republic of China. Minimum spanning tree of 12 W135 isolates typed by multilocus

sequence typing (MLST). Clustering of MLST profiles was performed by using a categorical coefficient. MLST types are indicated by circles. The size of each circle is proportional to the number of isolates with this particular type. Numbers (5, 6, and 7) indicate number of different loci between 2 MLST types. Thick solid lines connect types that differ at 1 locus and thin dotted lines connect types that differ at 2 loci. The color of each circle indicates types that belong to the same complex. MLST complexes were assigned if 2 neighboring types did not differ at >1 locus and if ≥2 types fulfilled this criterion. ST, sequence type.

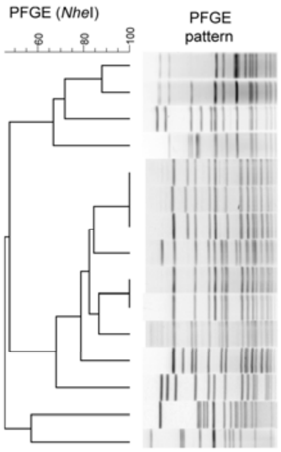

Strain code	Year of isolation	Province of isolation	Case or carrier	Site of isolation	MLST type	MLST complex	PorA type
450801	2008	Guangxi	Patient (case 3)	CSF	ST-11	ST-11 complex/ET-37 complex	P1.5,2
450802	2008	Guangxi	Close contact (case 3)	TS	ST-11	ST-11 complex/ET-37 complex	P1.5,2
440728	2007	Guangdong	Patient (case 2)	CSF	ST-11	ST-11 complex/ET-37 complex	P1.5,2
350601	2006	Fujian	Patient (case 1)	CSF	ST-11	ST-11 complex/ET-37 complex	P1.5,2
360245	1980	Jiangxi	Healthy carrier	TS	ST-5591	ST-174 complex	P1.21,16
360248	1980	Jiangxi	Healthy carrier	TS	ND		ND
360253	1980	Jiangxi	Healthy carrier	TS	ST-5591	ST-174 complex	P1.21,16
360258	1980	Jiangxi	Healthy carrier	TS	ND		ND
360492	1979	Jiangxi	Healthy carrier	TS	ST-5591	ST-174 complex	P1.21,16
360493	1979	Jiangxi	Healthy carrier	TS	ST-5591	ST-174 complex	P1.21,16
650418	1985	Xinjiang	Healthy carrier	TS	ND		ND
360257	1980	Jiangxi	Healthy carrier	TS	ST-5663	ST-174 complex	P1.21,16
R29057 (France)					ST-174	ST-174 complex	ND
130809	2008	Hebei	Healthy carrier	TS	ST-3436	ST-4821 complex	ND
440550	2005	Guangdong	Healthy carrier	TS	ST-5587	Single	P1.7-1.1

Figure 3. Distribution and molecular characterization of *Neisseria meningitidis* W135 isolates from case-patients, People's Republic of China. Cluster analysis of 15 strains base on pulsed-field gel electrophoresis (PFGE) patterns. Clustering was performed by using the Dice coefficient and a 1.2% optimization setting. The dendrogram was generated by using the unweighted pair group method using averages. MLST, multilocus sequence type; PorA, outer membrane protein; CSF, cerebrospinal fluid; ST, sequence type; TS, throat swab; ND, not determined.
